# Identification of a piscine reovirus-related pathogen in proliferative darkening syndrome (PDS) infected brown trout (*Salmo trutta fario*) using a next-generation technology detection pipeline

**DOI:** 10.1371/journal.pone.0206164

**Published:** 2018-10-22

**Authors:** Ralph Kuehn, Bernhard C. Stoeckle, Marc Young, Lisa Popp, Jens-Eike Taeubert, Michael W. Pfaffl, Juergen Geist

**Affiliations:** 1 Unit of Molecular Zoology, Department of Zoology, Technical University of Munich, Freising, Germany; 2 Department of Fish, Wildlife and Conservation Ecology, New Mexico State University, Las Cruces, NM, United States of America; 3 Aquatic Systems Biology Unit, Department of Ecology and Ecosystem Management, Technical University of Munich, Freising, Germany; 4 Fachberatung für Fischerei Niederbayern, Bezirk Niederbayern, Landshut, Germany; 5 Department of Animal Physiology and Immunology, Technical University of Munich, Freising, Germany; CNRS UMR7622 & University Paris 6 Pierre-et-Marie-Curie, FRANCE

## Abstract

The proliferative darkening syndrome (PDS) is an annually recurring disease that causes species-specific die-off of brown trout (*Salmo trutta fario*) with a mortality rate of near 100% in pre-alpine rivers of central Europe. So far the etiology and causation of this disease is still unclear. The objective of this study was to identify the cause of PDS using a next-generation technology detection pipeline. Following the hypothesis that PDS is caused by an infectious agent, brown trout specimens were exposed to water from a heavily affected pre-alpine river with annual occurrence of the disease. Specimens were sampled over the entire time period from potential infection through death. Transcriptomic analysis (microarray) and RT-qPCR of brown trout liver tissue evidenced strong gene expression response of immune-associated genes. Messenger RNA of specimens with synchronous immune expression profiles were ultra-deep sequenced using next-generation sequencing technology (NGS). Bioinformatic processing of generated reads and gap-filling Sanger re-sequencing of the identified pathogen genome revealed strong evidence that a piscine-related reovirus is the causative organism of PDS. The identified pathogen is phylogenetically closely related to the family of piscine reoviruses (PRV) which are considered as the causation of different fish diseases in Atlantic and Pacific salmonid species such as *Salmo salar* and *Onchorhynchus kisutch*. This study also highlights that the approach of first screening immune responses along a timeline in order to identify synchronously affected stages in different specimens which subsequently were ultra-deep sequenced is an effective approach in pathogen detection. In particular, the identification of specimens with synchronous molecular immune response patterns combined with NGS sequencing and gap-filling re-sequencing resulted in the successful pathogen detection of PDS.

## Introduction

For years, a suspicious species-specific die-off of brown trout (*Salmo trutta fario*) has been reported from pre-alpine river systems in Austria, Southern Germany, and Switzerland resulting in drastically decreased population densities in the impacted regions [1, 2]. In the most severely affected areas, no viable populations of brown trout remain and all attempts to restock brown trout in these places have failed due to the persistence of the annual die-off [1]. Since affected brown trout develop a black pigmentation on the skin before their death, the disease was named “Schwarze Bachforelle Phänomen” in German [1], which translates into “Black Trout Phenomenon”, equivalent to “Proliferative Darkening Syndrome” (PDS) [3]. Recently it was suggested that this disease could be primarily the result of immune suppression caused by a combination of temperature variation and UV-radiation but clear evidence on the causes could not be found [2]. It was also hypothesized that there is a strong link between the Proliferative Kidney Disease (PKD), caused by the parasite *Tetracapsuloides bryosalmonae*, and PDS [4].

In the affected river sections first external signs of PDS in brown trout include behavioral changes (decreased appetite and increasing listlessness), followed by emaciation, exophthalmia, gasping and the development of black sub-cutaneous spots [5] observable in the late summer (mid-August to late September). After the onset of external signs of PDS, affected individuals often die within hours while the cumulative die-off of a PDS-exposed brown trout population occurs over a time span of 2–3 weeks at a mortality rate of nearly 100% [1]. Interestingly, die-offs are only observed in late summer and only if brown trout have already been exposed to water from the PDS-affected river section in late spring, specifically between the beginning of May and the end of June [6]. It thus appears likely that brown trout already become exposed to the causative agent of PDS during spring, which then irreversibly leads to their die-off in the late summer [6]. Between mid-July to August, histopathological changes take place in several internal organs, predominantly in the liver as well as in the spleen and kidney (to a lesser degree). The initial histopathological changes in the liver include inflammation, appearance of multifocal lesions and hepatocyte degeneration. The kidney is characterized by lymphocyte proliferation, whereas the spleen becomes at the same time enlarged and depleted of lymphocytes, specifically of B-cells. As the disease progresses, hemorrhaging of the liver, kidney and spleen, multifocal necrotic lesions throughout the liver and spleen, nephrosis of the kidney as well as white plaque formations on the liver appear [5]. The course of PDS can be divided into three stages: (i) The initial stage following infection or contact with the causative agent, with no external signs of PDS (phenotypically healthy) and no pathological changes in internal organs; (ii) The clinical stage with no external signs of PDS (phenotypically healthy) but with pathological changes in internal organs, and (iii) the symptomatic stage with external signs of PDS (phenotypically sick) and severe pathological changes in internal organs terminated by the death of the organism.

Identification and management of diseases in salmonid fishes is particularly important due to their great ecological and economic importance. For instance, salmon and trout are among the most important finfish in aquaculture in Europe and America [7]. In addition, salmonids also play an important role in recreational fisheries worldwide which is underlined by the active introduction of salmonids into areas outside their original distribution range (e.g. New Zealand, South Africa, and South America). Consequently, knowledge on salmonid diseases is not only essential in understanding their impacts on the level of individuals and populations, but also in avoiding possible global spread.

The origin of a number salmonid diseases is still unknown [8]. Next generation technologies (high-throughput sequencing and high-throughput gene expression profiling) and bioinformatics applications are increasingly used to improve detection and the mechanistic understanding of infectious diseases and their outbreaks in fishes [9]. High throughput gene expression profiling and high-throughput sequencing are suited for the task of systematic virus discovery [10]. Microarrays have been successfully used in humans for detection of known and novel pathogens including their variants [11, 10, 12]. Next-generation sequencing has been particularly useful to aid human virus discovery by generating hundreds of thousands to millions of reads per run [13] and by allowing identification of novel virus even in exceedingly low titers. Enhanced bioinformatics packages have the potential to allow non-specialists in bioinformatics to detect and assemble viral genomes from deep sequence data-sets [14, 15]. However, to date only few studies have applied these tools in the context of gene expression profiling in fish (but see e.g. [16]), and no study has yet tested the usefulness of such approaches in clarifying the reason for the spurious die-off of brown trout in the Alpine region.

The objective of this study was to identify the cause of “Proliferative Darkening Syndrome” (PDS) and to characterize its etiology in brown trout (*Salmo trutta*) using a next-generation technology detection pipeline based on high-throughput sequencing and high-throughput gene expression profiling. We specifically hypothesized that the PDS is caused by an infectious agent and that the approach of first screening immune responses along a timeline to then identify synchronously affected stages in different specimens which subsequently are ultra-deep sequenced is an effective approach in pathogen detection. In a first step, brown trout were exposed to water from a heavily affected river with annual occurrence of the disease in order to generate tissue samples from spring to late summer (i.e. spanning over the entire time period from potential infection through death). Holistic transcriptomic analysis (microarray) and validation by RT-qPCR assays were conducted to reveal immune response of specimens during the infection period and to characterize individual variation of gene expression profiles. Next-generation sequencing of individuals and bioinformatics processing of generated reads enabled gap-filling intensive Sanger re-sequencing of the identified pathogen genome and determination of its taxonomic position.

## Material and methods

### Study design

**Fig 1:**
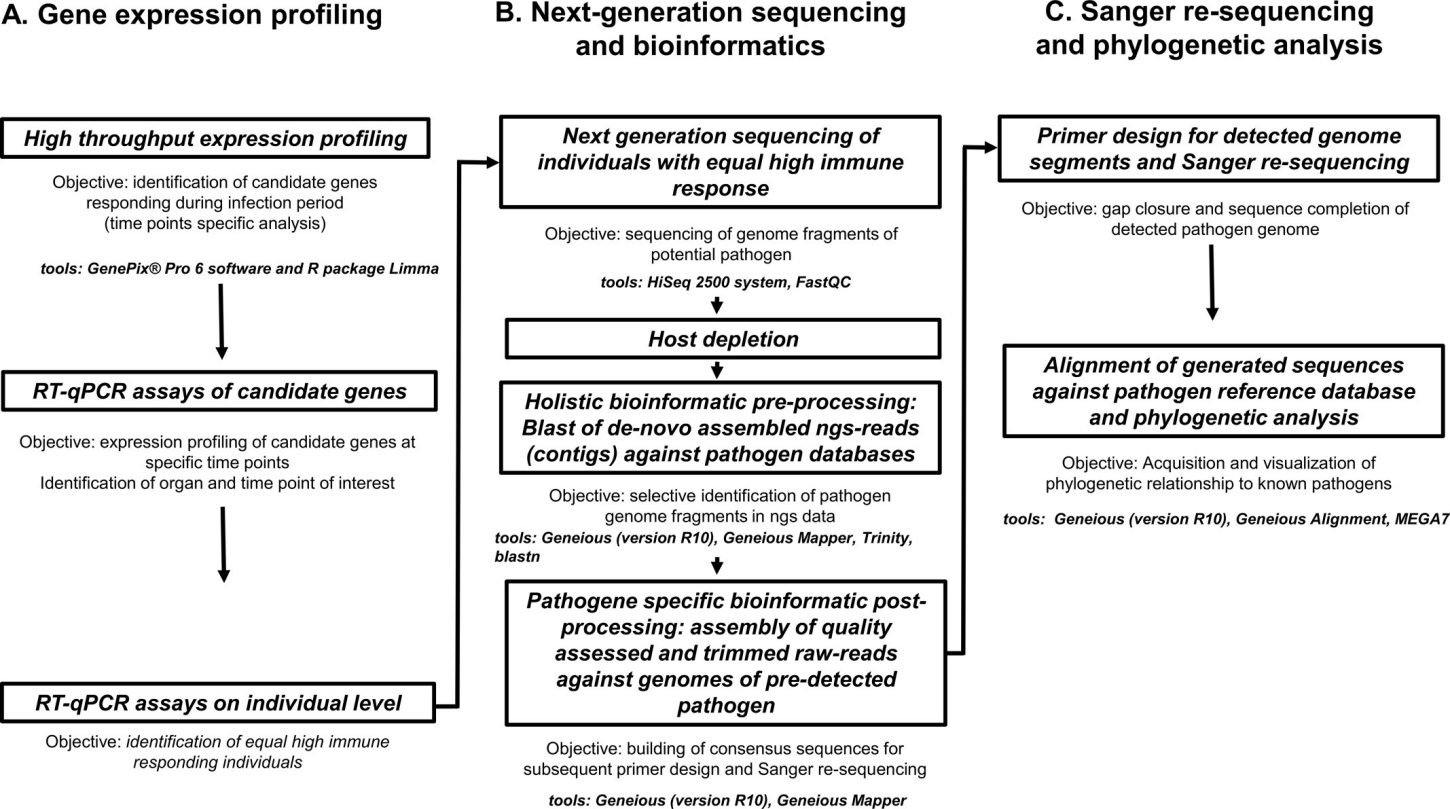
Schematic diagram visualizing the detection pipeline for an unknown pathogen in a non-model species in natural populations. Core steps in the workflow including the used tools from high throughput expression profiling to final phylogenetic analysis are displayed.

Our study design was primarily based on a comparison of brown trout exposed to PDS-affected river water and a control group exposed to spring water within the same area. Specimens were kept under otherwise similar conditions over a time period of 15 weeks covering the complete time window from possible first pathogen contact until die-off (i.e. from May 2008 through September 2008). Liver tissue from three specimens per group was sampled every day during the whole duration of the experiment and subsequently used for RNA extraction. Since no target pathogen was known at the beginning of the study, we chose a detection approach that did not target a specific pathogen in order to not pre-exclude any possible cause. For pathogen detection and a characterization of the chronology of immune response on mRNA transcriptome level, three different approaches were used: (A) *gene expression profiling*: Transcriptomic analysis (microarrays) of mRNA from liver tissue was used to monitor the chronology of immune response of individual specimens throughout the whole experiment. This resulted in the identification of immune response candidate genes (IRGs) responding to the infection. RT-qPCRs of IRGs enable sophisticated statistical analysis by using biological and technical replicates to identify specimens with synchronous response pattern; (B) *next-generation sequencing and bioinformatics*: cDNA from a selection of specimens with synchronous immune response were ultra-deep sequenced on an Illumina HiSeq 2500 next-generation sequencing platform following deep bioinformatics processing. This resulted in identifying the pathogen genetic signal from the comparison between host genome data, the ultra-deep sequencing data of infected specimens and the generated pathogen databases; (C) *Sanger re-sequencing and phylogenetic analysis*: In order to complete the genetic information of the detected pathogen, primers matching the processed sequence reads were designed for subsequent amplification and Sanger re-sequencing of the gaps in pathogen cDNA. The pathogen was then taxonomically and phylogenetically classified by comparing its sequence data with all available pathogen databases (Fig 1).

### Maintenance of specimens, exposure and sampling

On May 29, 2008, brown trout (*Salmo trutta fario*) of the same age class (1+) with an individual weight ranging between 25–85 grams were obtained from a single hatchery (Schwäbischer Fischereihof Salgen, Fachberatung für Fischerei Schwaben, Germany) and randomly allocated to one of two different stations that are both located along the Iller river, named here the control station (location near Oberstdorf, Germany; n = 70) and the experimental station (location near Kempten, Germany; n = 500). At both stations brown trout were held in tanks (two tanks with a density of 0.014m^3^ / fish at the experimental station and four tanks with a density of 0.017m^3^ / fish at the control station) that were supplied with water from the Iller river in a flow-through system. Over a 15 year average, the mean water temperature difference between control and experimental station is 0.6°C with a mean of 7.5 and 8.1°C, respectively (Bayerisches Landesamt für Umwelt; Gewässerkundlicher Dienst, www.gkd.bayern.de). All brown trout were treated with 0.4ml Baytril (Bayer Animal Health GmbH, Leverkusen, Germany) per kg of body weight after being transferred to their respective holding tanks in order to ensure the health of the brown trout at the start of the experiment. Over the course of the experiment brown trout were fed twice a week with Ecolife trout chow (BioMar, Brande, Denmark) using an amount corresponding to 1% of body weight. The experimental station is located roughly 40 km downstream from the control station and is separated by three anthropogenic transverse structures, two of which are impassable for fish. In the downstream Iller river section by Kempten, where the second station was located, PDS has been observed regularly and previous exposure experiments performed at the experimental station have confirmed that brown trouts exposed to local Iller water by Kempten suffer from PDS [5]. In contrast, no PDS event has ever been reported to have occurred at the up-stream control station by Oberstdorf. Additionally, an inventory was conducted at both locations. At the control station a healthy brown trout population was found to exist in the Iller River which is in contrast to the experimental station where no brown trout with PDS were documented during the inventory.

Sampling at experimental stations started on May 29, 2008, which was also the day on which the specimens were transferred to their exposure tanks (referred to as 0 day post exposure; d.p.e.), and ended on the 5th of September 2008. Three specimens, which showed no external signs of PDS (phenotypically healthy), were sampled each day (always at 2pm). Individuals were anaesthetized by a blow to the head and liver tissue was immediately harvested from sacrificed specimens, snap-frozen in liquid nitrogen and subsequently stored at -80°C until further processing. The liver was chosen as the organ of interest for this study as it is the most severely impacted organ during PDS and the pathological changes occurring in the liver are considered cardinal signs of PDS (hepatocyte degeneration, multifocal necrotic lesions, and white plaque formation).

### RNA extraction

Three liver samples of each day were homogenized by using the TissueRuptor homogenizer (Qiagen GmbH, Hilden, Germany) and lysed in QIAzol lysis reagent (Qiagen GmbH, Hilden, Germany). The RNA isolation was conducted according to the manufacturer’s handbook. Total RNA was quantified by Nanodrop ND-1000 (PeqLab, Erlangen, Germany) and RNA purity and absence of inhibitors was determined by spectrophotometric readings 260/280 nm and 260/230 nm absorption ratios. The qualitative RNA integrity was verified via RNA Integrity Number (RIN) measured by capillary electrophoresis measurements using the Bioanalyzer 2100 (Agilent Technologies).

### High throughput expression analysis, Microarray

For microarray analysis, the cGRASP 32K salmonid cDNA array [17] was used. The experiment was designed to fully comply with MIAME guidelines. Analyses were performed using a direct comparison two-channel design in which equimolar amounts of liver of one brown trout from both treatment and control group were co-hybridized on the same microarray. For the 14 time points (7, 14, 21, 28, 35, 42, 49, 56, 63, 70, 77, 84, 91, and 98 d.p.e.) microarray co-hybridizations were repeated in triplicate (n = 3) and included one dye-swap in order to reduce dye-bias. Hybridization processes were implemented according to the Genisphere Array 50 Protocol (revised version 5) (The Consortium for Genomic Research on All Salmon Project; [18]). Spot identification, intensity quantification and quality control were carried out with the GenePix Pro 6 software (Molecular Devices GmbH, Biberach, Germany). Analysis of the resulting GenePix files (*.gpr) were carried out with the open source R software package Linear Model for Microarray Data (Limma) [19] and the red and green intensities (RGlists) were background adjusted using the Kooperberg model-based correction [20]. Corrected RGlists were normalized within arrays by the Loess method followed by normalization between arrays using the scale method [21]. Significantly regulated genes (Benjamini and Hochberg's method) were screened for genes known for immune relevance in fish species [22, 23, 24, 25]. Reference genes were determined with NormFinder software [26]. The Microarray data set was submitted to NCBI's Gene Expression Omnibus (GSE70257). Hierarchical clustering with multiscale bootstrap resampling was performed with all significantly differentially regulated features using the pvclust package [27] in R [28].

### RT-qPCR of immune response candidate genes (IRGs)

Based on the microarray results, gene specific primer pairs were designed for significantly up-regulated genes and three non-regulated reference genes using Primer3 software [29]. Liver total RNA of three specimens sampled per time point (7, 14, 21, 28, 35, 42, 49, 56, 63, 70, 77, 84, 91, and 98 d.p.e.) were pooled in equimolar amounts and used (1 μg RNA in total) for RT-qPCR validation assays. After treatment with RNase-free DNase I (Thermo Scientific, Life Technologies GmbH, Darmstadt, Germany) and reverse transcription with the High Capacity cDNA Reverse Transcription Kit (Applied Biosystems, Life Technologies GmbH, Darmstadt, Germany), PCRs were performed on the 7500 Fast Real Timer PCR system (Applied Biosystems, Life Technologies GmbH, Darmstadt, Germany) using 5X HOT FIREPol EvaGreen qPCR Mix plus Rox (Solis BioDyne, Tartu, Estonia) with the following cycling conditions: Holding at 50°C for 20 seconds and continued for 10 minutes at 95°C followed by 40 cycles of 95°C for 30 seconds and a primer-specific annealing temperature (summarized in Table 1) for 30 seconds, amplification at 72°C for 30 seconds and a primer specific fluorescence measurement temperature for 30 seconds to ensure product-specific quantitation. qPCR efficiency was determined for all target gene primer pairs by 10x dilution of starting total RNA, with 5 dilution steps each in duplicates. BestKeeper applet [30] was used to analyze the expression stability of three candidate reference mRNAs.

**Table 1:** Summary of sequenced genome segments (λ1, λ2, λ3, μ2, μ1, μNS, σ3, σ2, σNS and σ1) of the virus detected in *S*. *trutta* (PRV Ger) and genetic similarity to piscine virus sequences detected in *S*. *salar*, *O*. *mykiss* and *O*. *kisutch* from Norway, Canada, Japan and Chile grouped in genotype cluster Ia, Ib, II and PRV2 according to Takano et al. [40] with additional information from GenBank entries (PRV3); Segment (Seg.), name of the segment (Name), fragment length in base pairs of the segments sequenced in this study (Seq. (bp)), segment function, sequence coverage of segments (in percent) (Ref. Seq.), and pairwise identity of sequenced fragment and corresponding reference sequence (P.I.). Segment IDs were assigned according to the nomenclature used by Palacios et al. (2010) [37]. (Accession numbers of reference segments are given in S2 Table).

Seg.	Name	Seq. (bp)	Function	PRV Ger vs. Genotype Ia (n = 2)		PRV Ger vs. Genotype Ib (n = 3)		PRV Ger vs. PRV2 (n = 1)		PRV Ger vs. PRV3 Chile (Genotype II) (n = 1)		PRV Ger vs. PRV3 Nor (Genotype II) (n = 1)	
				Ref. Seq (%)	P.I. (%)	Ref. Seq. (%)	P.I. (%)	Ref. Seq. (%)	P.I. (%)	Ref. Seq. (%)	P.I. (%)	Ref. Seq. (%)	P.I. (%)
**L1**	λ1 (Core shell)	1514	Helicase	38.85	78.35	38.93	78.43	38.80	75.80	38.20	98.40	39.40	97.30
**L2**	λ2 (Core turret)	800	Guanylyl-transferase	20.35	81.40	20.37	81.13	20.30	74.00	20.20	99.50	20.70	97.40
**L3**	λ3 (Core RdRp)	2636	RNA-dependent RNA polymerase	67.45	79.35	67.33	79.70	67.30	76.20	65.70	99.40	68.30	97.70
**M1**	μ2 (Core NTPase)	1220	Minor inner capsid protein	51.30	79.45	51.53	78.50	51.20	72.70	51.40	99.80	53.40	97.00
**M2**	μ1 (Outer shell)	1228	Outer capsid protein. membrane penetration	56.70	81.00	56.77	80.93	56.40	75.90	56.10	99.20	58.00	97.20
**M3**	μNS (NS factory)	629	Non-structural protein	26.25	81.90	26.37	82.30	26.20	65.80	25.70	98.60	27.80	98.40
**S1**	σ3 (Outer clamp)	767	Outer capsid protein. zinc metalloprotein	71.25	81.90	72.07	80.67	71.00	73.30	68.20	99.50	77.20	96.60
**S2**	σ2 (Core clamp)	1172	Inner capsid protein	88.60	79.60	89.10	79.93	88.10	69.60	87.00	99.50	92.90	99.50
**S3**	σNS (NS RNA)	699	Nonstructural protein	61.45	87.90	62.07	87.97	61.20	78.00	61.60	99.70	65.60	97.90
**S4**	σ1 (Outer fiber)	301	Virus attachment protein	29.10	90.00	29.40	89.80	29.00	71.80	28.60	100.0	30.50	99.70
			Mean	51.13	82.09		51.39	81.94		50.95	73.31		50.27	99.36	53.38	97.87

In order to screen for samples with similar or equal high immune response, established RT-qPCR assays were used to quantify the gene expression of selected IRGs on the individual level for 30 liver samples between 78 and 89 d.p.e. (three samples per day). RT-qPCRs were carried out as described above. The relative expression ratio (R) of selected IRGs was calculated using the efficiency (E) adjusted ΔΔCt method as described by Pfaffl [31]. Entire RT-qPCR workflow was performed according to the MIQE guidelines [32].

A nonparametric multidimensional scaling (NMDS) plot was created in order to display the Euclidean distance relationships among gene expression profiles of the selected genes associated with respective immune responses from individual liver samples. This indirect gradient analysis approach produces an ordination-based (distance or dissimilarity) matrix and projects the data into a Euclidean space. Pairwise dissimilarity of individual expression profiles can consequently be shown in a two-dimensional space.

### Next generation sequencing (Ultra-deep transcriptome sequencing) and bioinformatic pipeline

For next generation sequencing (NGS), from samples of individuals with comparably high immune response (according to the NMDS) strand-specific rRNA-depleted RNA-seq libraries were prepared using the Ovation Universal RNA-Seq System (NuGen Technologies, Leek, Netherlands) following the manufacturer’s specifications in combination with 324 InDA-C primers designed by NuGEN to target salmonid 18S and 28S rRNA transcripts for depletion. Sequencing libraries were quantified, pooled in equimolar concentration and sequenced on the next-generation sequencing platform Illumina HiSeq 2500 (Illumina, San Diego, CA, USA) producing 2 × 100-nucleotided single-end reads. For quality assessment and trimming, the raw reads were screened with FastQC (http://www.bioinformatics.babraham.ac.uk/projects/fastqc/) and the FASTX toolkit (http://hannonlab.cshl.edu/fastx_toolkit/index.html).

### Holistic bioinformatic pre-processing

In order to identify potential pathogens in the NGS data set, two custom blast databases were created and screened contigs were aligned against them. The first database was constructed from all viral nucleotide sequences obtainable from NCBI (https://www.ncbi.nlm.nih.gov/) (txid10239[Organism:salmonids] AND virus[filter]) (date of search: 11/2016) and is termed the virus database (vDB, 2,252,825 sequences). The second database was constructed using all ribosomal RNA sequences available within the SILVA SSU/LSU 132 datasets (date of search: 11/2016) and is termed the silva database (sDB; 4,985,791 small subunit (16S/18S) and 563,332 large subunits (23S/28S)). For host depletion, quality assessed and trimmed reads were first aligned to the *Salmo salar* transcriptome (109,584 sequences; NCBI Assembly ICSASG_v2) using Geneious (version R10) [15] with the medium/fast sensitivity setting of Geneious Mapper saving unused reads. Unused reads were de novo assembled to generate contigs using the Trinity software [33] with default parameters. Contigs were afterwards aligned against the vDB and sDB databases using blastn with the following parameters: -evalue 1E-040 -word_size 8 -gapopen 3 -gapextend 2 -reward 1 -penalty -1) (ftp://ftp.ncbi.nlm.nih.gov/blast/executables/blast+/).

### Pathogene specific bioinformatic post-processing

The quality assessed and trimmed reads were assembled against a piscine reovirus reference database (prvrDB). prvrDB was constructed using all available full genomes of piscine reovirus (PRV) (main result of the pre-processing blast analysis) from NCBI (date of search: 06/2018). The read assembly to reference (eight full PRV genomes) was conducted in Geneious (version R10) using the medium/fast sensitivity setting. Hits on references were used to build consensus sequences in order to design primer to fill sequence gaps by performing intensive Sanger sequencing.

### Intensive Sanger re-sequencing of the pathogene genome and phylogenetic analysis

Primers were designed for detected PRV genome segments using the Primer3 software [29]. PCRs were performed in a total volume of 12 μl with the following components: 20 ng of DNA, 0.3μM of each primer, 0.2 mM of each dNTP (Solis BioDyne), 1.6–2.8 mM MgCl2 (Solis BioDyne,), 1 × PCR buffer (Solis BioDyne) and 0.5 U Taq DNA Polymerase (FIREPol, Solis BioDyne). PCR products were purified using a NucleoSpin Extract Kit (Macherey and Nagel, Düren, Germany) and sequenced in both directions by Sequiserve GmbH (Vaterstetten, Germany).

These generated nucleotide sequences (17 fragments; 117–1211 bp in length) were aligned against the reference database (prvrDB) using Geneious (version R10) [15]. For each PRV segment interspecific diversities were analyzed and a phylogenetic tree of all concatenated sequences was created. For this purpose Maximum Likelihood method implemented in MEGA7 [34] was used to determine the best substitution model and to construct the phylogenetic tree using the best-fit model GTR+G. Furthermore, interspecific diversity and phylogenetic analyses were conducted with published PRV sequences of salmonid species using a fragment of the S1 segment (date of search on NCBI: 06/2018). To construct the phylogenetic tree, the Maximum Likelihood method implemented in MEGA7 [34] with substitution model K2+G was used.

## Animal ethics

All work that involved experimental animals was conducted in strict accordance to German Tierschutzgesetz (§8a and §11 TierSchG) and followed both the Bavarian institutional and German national ethical guidelines. The experiments reported here were approved with the following license numbers: AZ 209.1/211-2531.2-19/02 and AZ 568-1/2. and the animal welfare committee at TUM. After the experiments were completed, the remaining specimens (experimental and control group) were maintained at Schwäbischer Fischereihof Salgen, Fachberatung für Fischerei Schwaben, Germany.

## Results

### Exposure experiment

Brown trout at the experimental station exhibited the classical external signs of PDS (behavioral changes, emaciation, gasping and black sub-cutaneous spots) in the late summer (late August to beginning September) with nearly all specimens succumbing to PDS after 112 d.p.e.. First PDS symptoms were detected at 83 d.p.e.. Specimens maintained at the control location remained healthy without signs of PDS throughout the whole exposure experiment.

### Pathogen identification and phylogenetic relatedness

**Fig 2:**
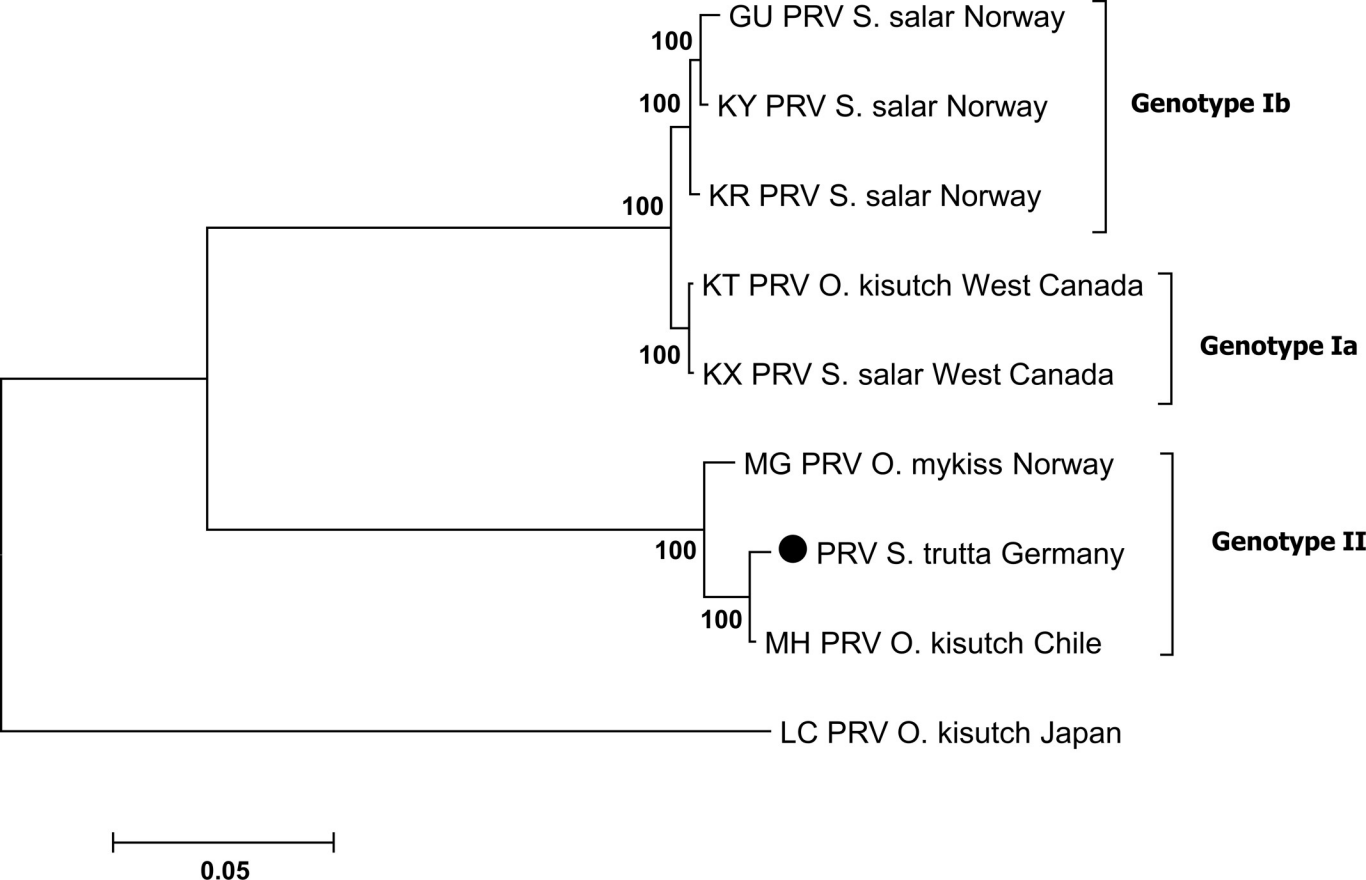
Phylogenetic analysis based on 51.0% of the genome sequence of the novel virus (λ1, λ2, λ3, μ2, μ1, μNS, σ3, σ2, σNS and σ1) and sequences of the piscine reovirus downloaded from the NCBI database. The scale bar (left below) refers to substitutions per amino acid sites. Numbers on the nodes represent the confidence limits (> 50%) estimated from 100 bootstrap replicates. The cluster definition (Genotype Ia, Ib, and II) is displayed according to Takano et al. [40]. The sequence of this study is symbolized by a solid circle.

Our approach of first screening immune responses along a timeline to identify synchronously affected stages in different specimens which then were subsequently ultra-deep sequenced revealed contigs similar to PRV genome fragments, pointing at a piscine reovirus as a likely causing agent of PDS. This was further confirmed by intensive gap-filling Sanger re-sequencing across these contigs where 51.0% of the total PRV reference genome was successfully sequenced (Table 1, designed primers are shown in S1 Table). More specifically, Sanger re-sequencing data was generated from all ten virus segments with coverage from 20% (L2, Core turret) to 93% (S2, Core clamp). The analysis of these identified sequence segments resulted in similarities between 73% and 100% to PRV and piscine orthoreovirus types detected previously in *S*. *salar* from Norway and West Canada [35, 36, 37, 38] in *Onchorhynchus kisutch* (Japan and North America) [39, 40], in *O*. *kisutch* from Chile (NCBI GenBank record, unpublished) and in *O*. *mykiss* from Norway (NCBI GenBank record, unpublished) (all GenBank accession numbers are provided in S2 Table). The phylogenetic clustering (Fig 2) of the concatenated segments of PRV is in accordance to Takano et al. 2016 [40] with the Genotype Ia, Ib, II and PRV-2. Particularly noteworthy is the close relatedness of the PRV genome found in specimens of *S*. *trutta* in Germany (in this study) and that found in *O*. *kisutch* from Chile and *O*. *mykiss* from Norway.

**Fig 3:**
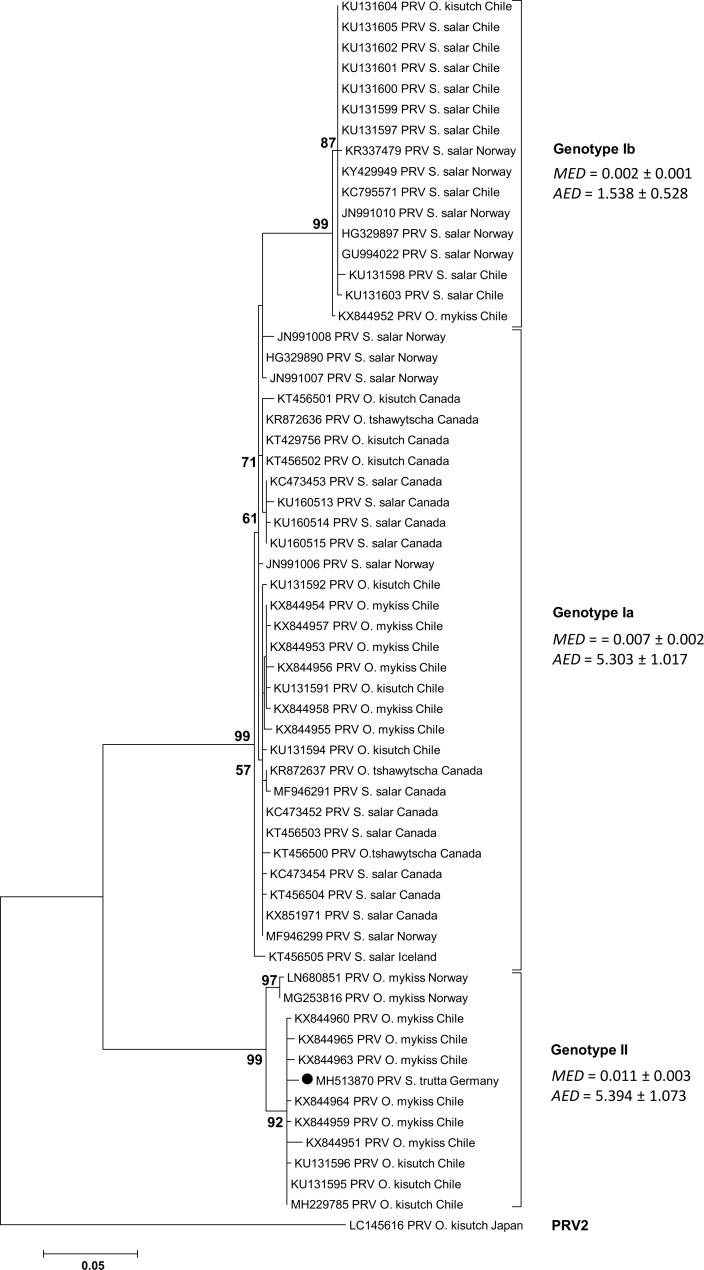
Phylogenetic analysis based on generated S1 sequence of the PRV-S1 from *S*. *trutta*, Germany, and sequences of the piscine reovirus downloaded from NCBI database. For each sequence the current GenBank accession number as well as the location where the virus was detected is shown. The scale bar (left below) refers to substitutions per amino acid sites. Numbers on the nodes represent the confidence limits (> 50%) estimated from 100 bootstrap replicates. The cluster definition (Genotype Ia, Ib, II and PRV 2) is displayed according to Takano et al. [40]. For every cluster the mean evolutionary diversity (*MED*) and the average evolutionary divergence (*AED*) was computed with MEGA7 [34]. The sequence of this study is symbolized by a solid circle.

The phylogenetic analyses with additional salmonid species using a fragment of the S1 segment revealed a clustering in the PRV genotype II with close relation the PRV-S1 fragment from *O*. *kisutch* and *O*. *mykiss* from Chile and Norway (Fig 3).

The approach taken in this study proved successful in detecting the likely causative organism of PDS. In order to obtain these results several steps of the next-generation pipeline and an exposure experiment were necessary. The results of these procedures are provided below.

### Microarray-based gene expression analysis

In total, 382 significantly regulated features were identified by the microarray analysis. The largest number of differentially regulated features as well as the majority of up-regulated features were observed at 84, 91 and 98 d.p.e., which corresponds to the time period after which the first individual in the experimental group succumbed to PDS (83 d.p.e.). According to the hierarchical clustering analysis, the 14 time points clearly separated into two distinct phases as suggested by the existence of two distinct clusters (S1 Fig). Cluster 1 contains the first 11 time points (7 to 77 d.p.e.) whereas Cluster 2 contains the last three time points (84 to 98 d.p.e.) with a strong expression activity in biological processes preceding the die-off. Seven genes associated with immune response in fish [22, 23, 24, 25] were identified as candidate genes for the single RT-qPCR analysis.

### RT-qPCR of immune response candidate genes (IRGs)

Primers were designed for the following genes: barrier-to-autointegration factor (*BAF*), C-C motif chemokine 19 precursor (*CCL19*), NOD-like receptor family CARD domain containing 5 (NLRC5), Interferon regulator factor 1 (*IRF-1*), Interferon alpha 1 (*IFNa1*), Interferon gamma (*IFN-g*), Major histocompatibility complex I (*MHC-I*) (Table 2). *60S* ribosomal protein *L28*, *40S* ribosomal protein S10 and Ubiquitin were used as multiple reference genes to calculate the relative expression ratio (R) of target mRNA using the efficiency adjusted ΔΔCt method as described by Pfaffl [31]. PCR efficiency for all primer pairs was 1.96±5.

**Table 2:** The seven selected genes associated with immune response in fish species (according to [21, 22, 23, and 24]) and the three reference genes: Function of immune relevant genes, designed RT-qPCR primers, product size (bp), annealing temperature (Ann. Temp.) and temperature for fluorescence acquisition (Fluor. Temp.).

Gene	Function	Primers (5' - 3')	Size (bp)	Ann. Temp. (°C)	Fluor. Temp. (°C)
Barrier-to-autointegration factor	early response gene to viral infections	F: gccgcaggctagaggagaag	116	57	80
*BAF*		R: ggcccctgtggtatttttca			
C-C motif chemokine 19 *CCL19*	recruiting monocytes, neutrophils, and other effector cells from vessels towards the focus of infection	F: ctcctgccaccaagaacaac	119	57	78
		R: acccacagcctttcagtgtc			
NOD-like receptor family CARD *NLRC5*	recognition of microbial pathogens	F: acctgacccatgaggatgga	217	57	82
		R: gcagcaagcccacaaaacat			
Interferon regulatory factor 1 *IRF-1*	modulating the expression of interferon genes	F: gctgctctgtgacagttgga	105	60	75
		R: gcacgatttaacaaaagagtggat			
Interferon alpha 1 *IFN-a1*	signaling pathways in response to pathogen infection or pathogen associated molecular pattern stimulation	F: acagcgaaacaaacagctattt	89	57	73
		R: gacacacgctctgcatactg			
Interferon gamma *IFN-g*	signaling pathways in response to pathogen infection or pathogen associated molecular pattern stimulation	F: actgaaagtccactataagatctc	370	57	83
		R: tggaacttaagggccagtttg			
Major histocompatibility complex I *MHC-I*	initiating CD8^+^ cytotoxic T cell-mediated cellular immunity	F: tgaagccatcaaaacaacca	138	60	75
		R: gagcaaagatcgaacatgtca			
*60S* ribosomal protein *L28*		F: catccgcaagagcaactaca	101	60	83
		R: cctcttcaccaccaccacac			
*40S* ribosomal protein *S10*		R: cccaagaagaaccgtattgc	121	60	80
		R: gaaggttgggcacgttctt			
Ubiquitin		F: cttcatcttgtgctgcgtct	147	60	82
		R: acacttcttcttgcggcagt			

**Fig 4:**
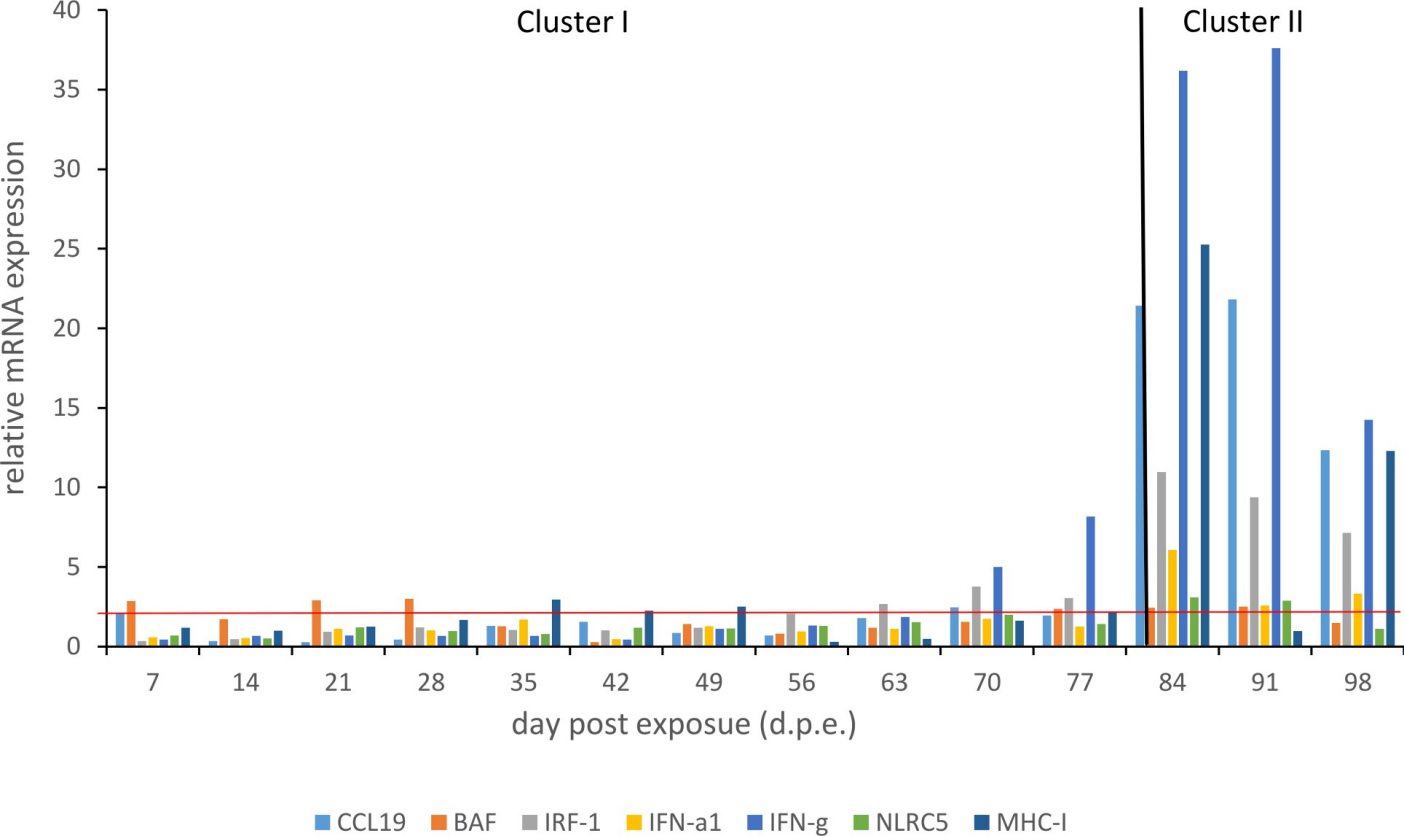
Gene expression profiles over experimental time course (7 to 98 d.p.e.) of genes involved in immune response: Barrier-to-autointegration factor (*BAF*), C-C motif chemokine 19 precursor (*CCL19*), NOD-like receptor family CARD domain containing 5 (*NLRC5*), Interferon regulator factor 1 (*IRF-1*), Interferon alpha 1 (*IFNa1*), Interferon gamma (*IFN-g*), Major histocompatibility complex I (*MHC-I*); result of the hierarchical clustering analysis based on microarray results (cluster I and cluster II).

**Fig 5:**
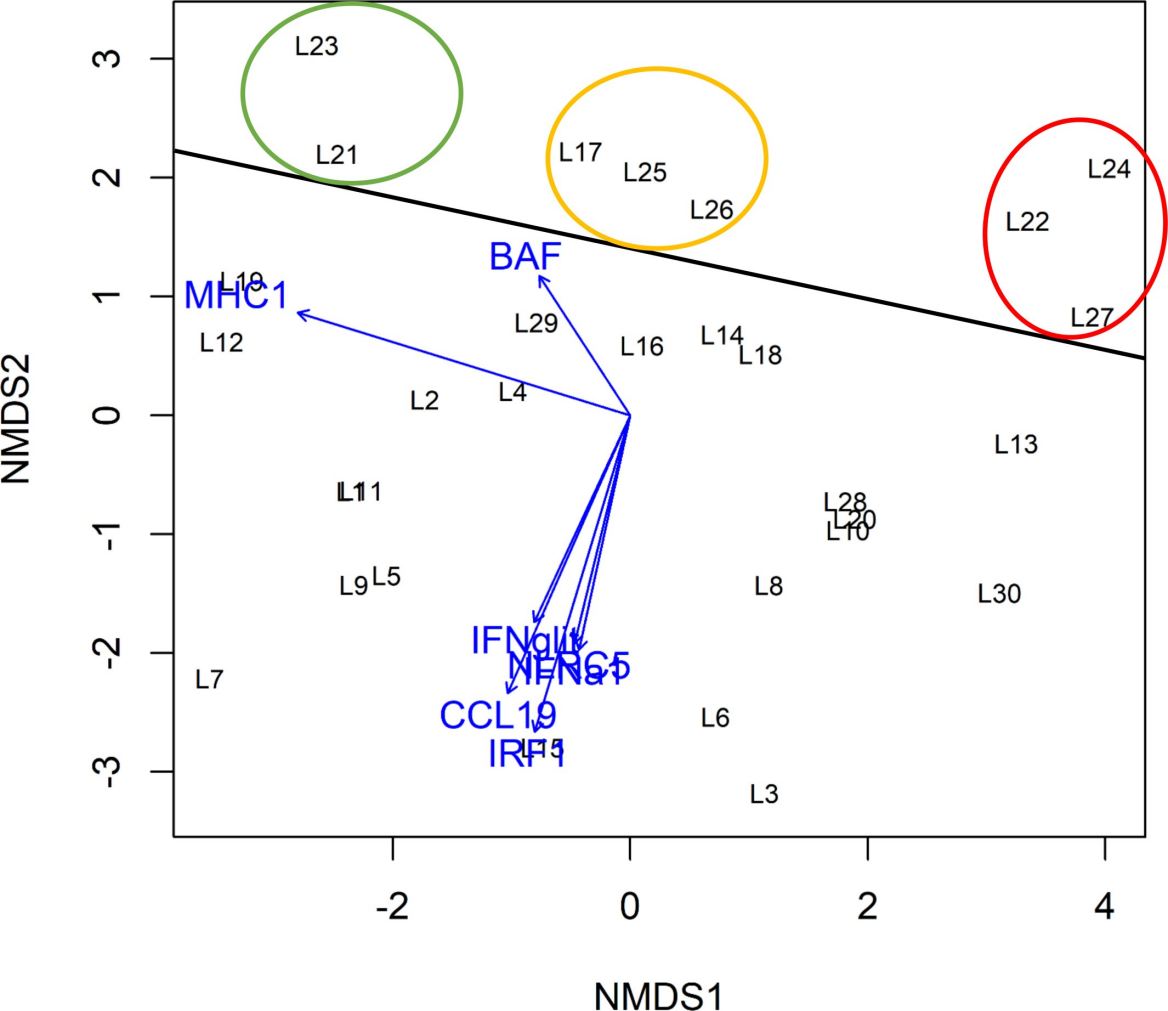
Result of the nonparametric multidimensional scaling (NMDS) with immune response genes (IRG) gene expression profiles of 30 liver samples (L1 to L30). The genes *BAF*, *CCL19*, *NLRC5*, *IRF-1*, *IFNa1* und *IFN-g* were most strongly upregulated in the samples L27, L21, L22, L23, L24, L25, L26 and L27 displayed above the black horizontal line. The circles refer to the degree of MHC-I regulation of those samples: strongly downregulated (green circle), slightly upregulated (orange circle) and strongly upregulated (red circle) according to IRG expression profiles.

RT-qPCRs of pooled liver samples revealed strong gene expression changes for the immune-relevant candidate genes. The highest response was evident in cluster II (84, 91 and 98 d.p.e.) for the genes *IFN-g*, *MHC-I* and *CCL19* with maximum values of 37.6, 25.3 and 21.8, respectively (Fig 4; S3 Table). Of particular interest is the transition from cluster I to cluster II where the immune response resulted in an exponential increase of gene expression. In order to screen for specimens of similar or equal high immune response, gene expression profiles of selected IRGs were analyzed for 30 liver samples (L1 to L30) between 78 and 89 d.p.e. (S4 Table). Expression profiles were displayed in a nonparametric multidimensional scaling (NMDS) plot (Fig 5). Dissimilarity between expression profiles of individual samples equates to distance in the NMDS plot.

Grouping of samples displays equal gene expression of the IRGs. Samples in the lower part of the plot showed low expression of *CCL19*, *IRF-1*, *IFNa1*, *IFN-g* and *NLRC5* (e.g. L3, L6 and L15), while the upper part of the plot contains samples with highest expression of these IRGs (L17, L21-L27). MHC-I gene expression was also different with highest values for samples L13, L22, L24; L27 and L30 (right side of the plot) clustering in three groups (colored circles) according to *MHC-I* expression. Samples L22, L24 and L27 showing equal high immune response for all IRGs were selected for subsequent next-generation sequencing.

### Next-generation sequencing, de novo assembly of transcriptome and annotation

Three high-quality sequencing libraries of the liver samples showing equal high immune response were sequenced on an Illumina Hiseq 2500 platform generating a total of 380 million 100bp single end raw reads. The NGS data set was submitted to NCBI's Sequence Read Archive (*SRP154201*). Of these reads, 89% were kept after quality control and trimming. Trimmed reads were mapped to the reference transcriptome (*S*. *salar*) and for the 269 million unused reads 1.2 million contigs were assembled. The alignment against the vDB of these contigs revealed four hits showing a high similarity with the piscine reovirus (PRV) (outer clamp segment (S1): 276 bp, 99%, 1E-134; core NTPase and outer shell segment (M1 and M2): 201 bp, 83%, 5E-50, segment L3: 213bp, 80%, 4E-46, core shell segment (S2): 204, 80%, 1E-42) and one contig was aligned to RV brown trout mRNA for polyprotein (203 bp, 86%, 5E-55). The alignment against the sDB showed similarity to several myxosporidan species *Chloromyxum truttae* partial 18S rRNA gene: 365 bp, 99%, P = 0; *Myxidium truttae* partial 18S rRNA gene: 269 bp. 100% p = 1E-133; *Sphaerospora truttae* partial 18S rRNA gene: 415 bp, 99%, p = 0: *Zschokkella nova* isolate M0289 28S large subunit ribosomal RNA gene: 396 bp, 97%. p = 0; *Chloromyxum cristatum* isolate M0178 28S large subunit ribosomal RNA gene: 212 bp, 93%, p = 1.00E-083; *Chloromyxum fluviatile* isolate 705 18S ribosomal RNA gene: 258 bp, 92%, p = 3E-96).

The assembling of the quality assessed and trimmed raw reads (337 million) against the prvrDB resulted in 289 hits on the ten segments of the full genomes of the PRV references. Amplification systems for all segments were designed for gap-filling intensive Sanger re-sequencing and phylogenetic analysis (see section *Pathogen identification and phylogenetic relatedness)*.

## Discussion

After many decades of speculation and numerous unsuccessful efforts to identify the reasons for the spurious brown trout die-off (PDS) in the Alpine region, this study is the first one to provide strong evidence for a piscine-related reovirus as the likely reason. The findings also demonstrate that the approach of first screening immune responses along a timeline to then identify synchronously affected stages in different specimens which subsequently were ultra-deep sequenced is an effective approach in pathogen detection, especially if any unknown possible causes of disease should not be pre-excluded. In particular, the identification of specimens with synchronous molecular immune response patterns and the sequencing and gap-filling approach in concert resulted in the successful pathogen detection of this reovirus with its very long incubation period extending over several months.

The used next-generation technology pathogen detection pipeline also has some drawbacks such as the costly effort of the pre-analysis of the expression patterns. The pre-analyses of gene expression is not a mandatory prerequisite, especially if the potential causes of disease can already be narrowed down to few well-defined pathogens initially. Reduced detection pipelines in addition with user-friendly bioinformatics software packages revealed also successful pathogen detection [14, 41]. However, the identification and grouping of synchronously affected stages in different specimens increases the effectiveness of the ultra-deep NGS and reduces the informatics requirements for the data analysis which can be challenging due to data file sizes [42]. NGS data sets generated for pathogen detection can be composed of mostly host-derived sequences and a minor, sometimes minute fraction of pathogen sequences that must be laboriously separated [43]. Since fishes are capable of reducing their viral load [44], virus sequences can occur at very low levels in hosts, i.e. in the sample which has to be analyzed [45]. As a certain proportion of sequences generated by NGS approaches remain uncharacterized because no similar sequences are available from gene banks, possible causatives can be overlooked. The verification of a relation between found pathogen and a specific disease can also be a difficult challenge, however the application of next-generation sequencing combined with bioinformatics approaches unraveled a large number of previously unknown pathogens of aquatic organisms and have significantly accelerated the ability to identify novel viruses of fish [46].

Koch’s postulate requires demonstration that an agent causes a disease, and that disease can be reproduced in a native host by inoculation with the agent propagated in culture following isolation from an affected host. Although fulfillment of this postulate is compelling evidence of causation, the criteria are sometimes extremely difficult to fulfil [37]. However, screening and the analysis of IRGs gene expression profiles provide indication that the detected pathogen of this study is the likely causative of the PDS in brown trout.

Conventional methods for virus detection, particularly PCR, serology, electron microscopy and virus culture have proven many times for identifying new viruses [10]. But all of these methods have limitations regarding systematic discovery of unknown pathogenic agents. Virus-specific PCR detection techniques offers high sensitivity, but presuppose precise knowledge of sequence data for primer design, which is not applicable for novel and unknown virus [47] as originally also the case in our study. Using sera of infected hosts enables to label virus in order to enhance detection in cell culture or electron microscopy, but high titers of labeled virus and specific viral antibodies are necessary [48, 49]. Electron microscopy is useful to detect new virus but the information is limited since only morphological information of the virus can be gained [48]. Many viruses cannot grow in culture or do not show characteristic cytopathic effects during growth [50]. This is especially the case for virus found in aquatic environments [51].

Although still being a multifaceted approach, our applied NGS based detection and identification pipeline does not need prior knowledge of the pathogen and its genome and it is suited for the task of systematic virus discovery. As demonstrated in this study, the NGS analysis of specimens with synchronous molecular immune response increase the pathogen detection success and the IRGs expression profiles provides incidences of the causative impact of the pathogen within the meaning of the Koch’s postulate.

The identified pathogen is closely related to virus of the Reoviridae family and a member of the PRV complex which is considered as a main disease problem in both Atlantic and Pacific salmonid species such as *S*. *salar*, *O*. *mykiss* and *O*. *kisutch* [40, 52]. Phylogenetic analysis of the pathogen genome found in this study and full genomes of known PRV revealed that the sequence similarity was between 73% and 82% to PRV and piscine orthoreovirus types detected in *S*. *salar* (Norway and West Canada) [35, 36, 37, 38] and *O*. *kisutch* (Japan and North America) [39, 40]. A similarity of 98% and 99% was revealed to a full genome of PRV detected in *O*. *mykiss* and *O*. *kisutch* in Norway and Chile (NCBI Database, unpublished). Comparison of the revealed S1 sequence with 59 published sequences and GenBank records of the PRV confirmed the results of the genomic approach. The phylogenetic analysis suggests a non-regional descent of this pathogen. An anthropogenic induced dispersal based on supraregional stocking is assumable, however a species specificity of the virus should also be taken into consideration.

The general importance, occurrence and etiology of PRV is well documented [53, 54, 36]. PRV has been described as a double-stranded RNA virus with ten nucleic acid segments (λ1, λ2, λ3, μ2, μ1, μNS, σ3, σ2, σNS and σ1) [37, 55] which is in concordance with our findings. So far, the presence of PRV has been confirmed in farmed and wild salmonid species from Northern Europe [52, 44], from the west coast of North America [45, 39, 36, 56] and from South America [57, 58] where salmonids are not native. Here we show the first evidence of PRV in the Alpine region of Central Europe as the likely causative organism of PDS. The high similarity of PRV of the Alpine region with virus specimens of Northern Europe and Southern America points to an anthropogenic transmission of this PRV via transfer and stocking of farmed and wild salmonids, which is in concordance with the findings of Garseth et al. [52]. The international, supraregional trading of salmonids due to their socioeconomic importance must increase awareness of the problem of pathogen spread which should result in an international risk assessment in the context of pathogen dissemination in aquaculture.

## Supporting information

S1 Fig Hierarchical clustering analysis of microarray analysis results. (DOCX)

S1 Table Designed primers for PRV genome segments detected in this study. (DOCX)

S2 Table GenBank accession numbers of PRV reference sequences and of PRV sequences (Germany; host: Salmo trutta) found in this study. (DOCX)

S3 Table RT-qPCRs results of pooled liver samples at 15 sampling time points (d.p.e.) for seven immune relevant genes. (DOCX)

S4 Table Gene expression of selected IRGs measured individually for 30 livers sampled between 78 d.p.e. and 89 d.p.e. (DOCX)
