# Supplementary material for: Identification of a piscine reovirus-related pathogen in proliferative darkening syndrome (PDS) infected brown trout (Salmo trutta fario) using a next-generation technology detection pipeline
Source: PLoS One. 2018 Oct 22;13(10):e0206164. doi: 10.1371/journal.pone.0206164 (PMC6197672; doi:10.1371/journal.pone.0206164)
Supplement: S1 Fig — (DOCX) [file pone.0206164.s001.docx]

**Supporting information S1 Fig.**


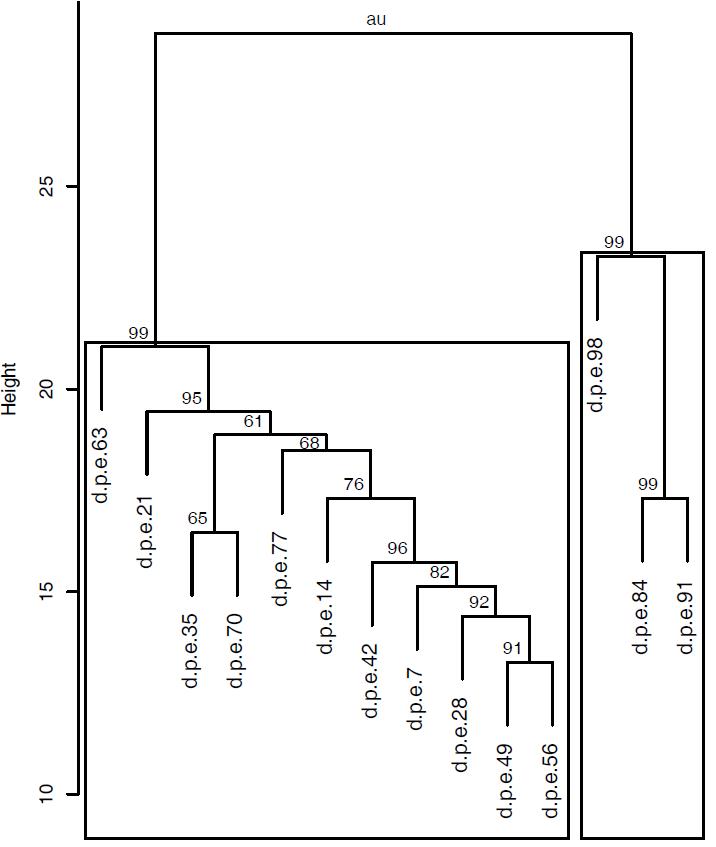


S1 Fig. Hierarchical clustering analysis of microarray analysis results
