# Supplementary material for: Identification of a piscine reovirus-related pathogen in proliferative darkening syndrome (PDS) infected brown trout (Salmo trutta fario) using a next-generation technology detection pipeline
Source: PLoS One. 2018 Oct 22;13(10):e0206164. doi: 10.1371/journal.pone.0206164 (PMC6197672; doi:10.1371/journal.pone.0206164)
Supplement: S1 Table — (DOCX) [file pone.0206164.s002.docx]

**Supporting information S1 Table**

S1 Table: Designed primers for PRV genome segments detected in this study

| segment | name | primer name | forward | reverse | length (bp) |
| --- | --- | --- | --- | --- | --- |
| L1 | λ1 |  |  |  |  |
|  |  | L1-1 | agacttgactgctgacgaca | ttcccaacttcgtctcacca | 359 |
|  |  | L1-2 | cgaagttgggaagatcgtct | acaatcctcacaacagctgc | 483 |
|  |  | L1-3 | gccgatcaagcttacgactc | gggttgatctccagcatg | 672 |
| L2 | λ2 |  |  |  |  |
|  |  | L2-1 | tggctacgctttatgggcta | caacgtcgcattccatctga | 792 |
|  |  | L2-2 | ctacgctttatgggctacgc | ggcttgatcaacgtcgcatt | 797 |
| L3 | λ3 |  |  |  |  |
|  |  | L3-1 | tgtcaaccaactacctgaagac | gacgcttctagtaccgacga | 599 |
|  |  | L3-2 | ctttctcggagtacctgcca | actcctctcaaccatccagc | 832 |
| M1 | µ2 |  |  |  |  |
|  |  | M1-1 | cgcaaaccgtgtttctagga | aaaaggttcactccacgcac | 1218 |
|  |  | M1-2 | gacaaccgttttctgctgcc | tgcctttctgactgcacaca | 306 |
| M2 | µ1 |  |  |  |  |
|  |  | M2-1 | attttgggtaactggcgacg | gtacaacgtactgccagggt | 541 |
|  |  | M2-2 | ccaccacctacactcaaca | tacaacgtactgccagggtg | 688 |
| M3 | µNS |  |  |  |  |
|  |  | M3-1 | actactacaatggctgaatca | tgcatgaatcctttgacgta | 629 |
| S1 | σ3 |  |  |  |  |
|  |  | S1-1 | taggacggcgacaactactg | tctaaggcgtcgcttagctt | 424 |
|  |  | S1-2 | tgctaaagttcatggtgcgag | tccttctgaccatgttcacca | 170 |
| S2 | σ2 |  |  |  |  |
|  |  | S2-1 | tctcgggtatttctgcctttt | ggtatagcgtgttggtccct | 117 |
|  |  | S2-2 | agggaccaacacgctatacc | ccgtgtatttgccgccatta | 1055 |
| S3 | σNS |  |  |  |  |
|  |  | S3-1 | gaacttcgatcttgggcgtc | ggaagcttcagaccatacgc | 699 |
| S4 | σ1 |  |  |  |  |
|  |  | S4-1 | ctcagcttaaccatgcatagct | ttcagtgaagcgaagttccg | 104 |
|  |  | S4-2 | cggaacttcgcttcactgaa | tgtcgtcaatctcaggacca | 217 |
