# Supplementary material for: Identification of a piscine reovirus-related pathogen in proliferative darkening syndrome (PDS) infected brown trout (Salmo trutta fario) using a next-generation technology detection pipeline
Source: PLoS One. 2018 Oct 22;13(10):e0206164. doi: 10.1371/journal.pone.0206164 (PMC6197672; doi:10.1371/journal.pone.0206164)
Supplement: S2 Table — (DOCX) [file pone.0206164.s003.docx]

**Supporting information S2 Table**

S2 Table: GenBank accession numbers of PRV reference sequences and of PRV sequences (Germany; host: *Salmo trutta*) found in this study

| **Segment** | **Accession** |  | **Segment** | **Accession** |  | **Segment** | **Accession** |
| --- | --- | --- | --- | --- | --- | --- | --- |
|  |  |  |  |  |  |  |  |
| **L1** | KY429945 |  | **M1** | KY429946 |  | **S1** | KY429949 |
|  | GU994013 |  |  | GU994017 |  |  | GU994022 |
|  | KR337473.1 |  |  | KR337476.1 |  |  | KR337479.1 |
|  | KX851982 |  |  | KX851976 |  |  | KX851971 |
|  | KT429750.1 |  |  | KT429753.1 |  |  | KT429756.1 |
|  | LC145610.1 |  |  | LC145612.1 |  |  | LC145616.1 |
|  | MH229778 |  |  | MH229779 |  |  | MH229785 |
|  | MG253809 |  |  | MG253810 |  |  | MG253816 |
|  | MH513858 (this study) |  |  | MH513866 (this study) |  |  | MH513870 (this study) |
|  | MH513859 (this study) |  |  |  |  |  |  |
|  | MH513860 (this study) |  |  |  |  |  |  |
| **L2** | KY429944 |  | **M2** | KY429947 |  | **S2** | KY429950 |
|  | GU994014 |  |  | GU994016 |  |  | GU994019 |
|  | KR337474.1 |  |  | KR337477.1 |  |  | KR337480.1 |
|  | KX851980 |  |  | KX851974 |  |  | KX851968 |
|  | KT429751.1 |  |  | KT429754.1 |  |  | KT429757.1 |
|  | LC145608.1 |  |  | LC145613.1 |  |  | LC145614.1 |
|  | MH229777 |  |  | MH229780 |  |  | MH229783 |
|  | MG253808 |  |  | MG253811 |  |  | MG253813 |
|  | MH513861 (this study) |  |  | MH513867 (this study) |  |  | MH513871 (this study) |
|  |  |  |  | MH513868 (this study) |  |  | MH513872 (this study) |
| **L3** | KY429943 |  | **M3** | KY429948 |  | **S3** | KY429951 |
|  | GU994015 |  |  | GU994018 |  |  | GU994020 |
|  | KR337475.1 |  |  | KR337478.1 |  |  | KR337481.1 |
|  | KX851978 |  |  | KX851972 |  |  | KX851966 |
|  | KT429752.1 |  |  | KT429755.1 |  |  | KT429758.1 |
|  | LC145609.1 |  |  | LC145611.1 |  |  | LC145615.1 |
|  | MH229776 |  |  | MH229781 |  |  | MH229784 |
|  | MG253807 |  |  | MG253812 |  |  | MG253814 |
|  | MH513862 (this study) |  |  | MH513869 (this study) |  |  | MH513873 (this study) |
|  | MH513863 (this study) |  |  |  |  | **S4** | KY429952 |
|  | MH513864 (this study) |  |  |  |  |  | GU994021 |
|  | MH513865 (this study) |  |  |  |  |  | KR337482.1 |
|  |  |  |  |  |  |  | KX851964 |
|  |  |  |  |  |  |  | KT429759.1 |
|  |  |  |  |  |  |  | LC145617.1 |
|  |  |  |  |  |  |  | MH229782 |
|  |  |  |  |  |  |  | MG253815 |
|  |  |  |  |  |  |  | MH513874 (this study) |
