# Supplementary material for: Identification of a piscine reovirus-related pathogen in proliferative darkening syndrome (PDS) infected brown trout (Salmo trutta fario) using a next-generation technology detection pipeline
Source: PLoS One. 2018 Oct 22;13(10):e0206164. doi: 10.1371/journal.pone.0206164 (PMC6197672; doi:10.1371/journal.pone.0206164)
Supplement: S3 Table — (DOCX) [file pone.0206164.s004.docx]

**Supporting information S3 Table**

S3 Table: RT-qPCRs results of pooled liver samples at 15 sampling time points (d.p.e.) for seven immune relevant genes

| d.p.e. | CCL19 | BAF | IRF | IFN-a1 | IFN-g | NLRC5 | MHC-I |
| --- | --- | --- | --- | --- | --- | --- | --- |
| 0 | 0.626797068 | 0.6000244 | 0.108877 | 0.2606 | 0.283254 | 0.170536 | 0.399206 |
| 7 | 2.052610891 | 2.8526644 | 0.348858 | 0.582142 | 0.425703 | 0.687467 | 1.171487 |
| 14 | 0.350372251 | 1.7232172 | 0.451173 | 0.515726 | 0.662726 | 0.506042 | 0.989618 |
| 21 | 0.269490073 | 2.9105659 | 0.91485 | 1.099978 | 0.69506 | 1.204126 | 1.245103 |
| 28 | 0.421197903 | 2.9838197 | 1.207761 | 1.018171 | 0.662542 | 0.957208 | 1.674938 |
| 35 | 1.302423131 | 1.2666954 | 1.041725 | 1.684791 | 0.661343 | 0.78575 | 2.94134 |
| 42 | 1.5514898 | 0.2804512 | 1.024894 | 0.443684 | 0.436554 | 1.1789 | 2.243524 |
| 49 | 0.847888687 | 1.4035388 | 1.187785 | 1.268735 | 1.117142 | 1.134926 | 2.498281 |
| 56 | 0.6870044 | 0.793666 | 2.057172 | 0.950086 | 1.308095 | 1.294083 | 0.301919 |
| 63 | 1.773680128 | 1.1833958 | 2.668046 | 1.11321 | 1.843473 | 1.53803 | 0.489263 |
| 70 | 2.466590911 | 1.5486029 | 3.755134 | 1.737255 | 4.990041 | 1.999964 | 1.620366 |
| 77 | 1.951665874 | 2.3635778 | 3.035251 | 1.235751 | 8.155845 | 1.405046 | 2.143152 |
| 84 | 21.42782021 | 2.4323794 | 10.95684 | 6.060167 | 36.17835 | 3.08693 | 25.25358 |
| 91 | 21.81242683 | 2.50259 | 9.365848 | 2.577068 | 37.61975 | 2.876511 | 0.975113 |
| 98 | 12.32363078 | 1.4723706 | 7.138091 | 3.329313 | 14.24297 | 1.105861 | 12.27673 |
