# Supplementary material for: Identification of a piscine reovirus-related pathogen in proliferative darkening syndrome (PDS) infected brown trout (Salmo trutta fario) using a next-generation technology detection pipeline
Source: PLoS One. 2018 Oct 22;13(10):e0206164. doi: 10.1371/journal.pone.0206164 (PMC6197672; doi:10.1371/journal.pone.0206164)
Supplement: S4 Table — (DOCX) [file pone.0206164.s005.docx]

**Supporting information S4 Table**

S4 Table: Gene expression of selected IRGs measured individually for 30 livers sampled between 78 d.p.e. and 89 d.p.e.

|  | CCL19 | BAF | NLRC5 | MHC1 | IFNa1 | IFNglit | IRF1 |
| --- | --- | --- | --- | --- | --- | --- | --- |
| **(1) T78-L-1** | -0.30390667 | 1.09503341 | 1.0738984 | 0.4077925 | -4.92992583 | -1.29618509 | -1.76412892 |
| **(2) T78-L-2** | -1.73503929 | 0.62811449 | 0.60877302 | 0.20576805 | -4.56511805 | -1.84155772 | -2.05574725 |
| **(3) T78-L-3** | -0.21075225 | -0.90109642 | 1.11316133 | -3.41595371 | -5.0376451 | 0.1324726 | -1.18239442 |
| **(4) T79-L-1** | -1.72817461 | 1.31980793 | 0.26986464 | -0.68121123 | -4.0274961 | -2.65384014 | -2.00374071 |
| **(5) T79-L-2** | -1.70821809 | -0.86438036 | 1.01327467 | 0.30487395 | -4.0363024 | -1.02721198 | -1.46739689 |
| **(6) T79-L-3** | -0.62028877 | -0.58932837 | 1.379558 | -2.91791718 | -4.02934258 | -1.3446947 | -0.9829069 |
| **(7) T80-L-1** | 0.59934508 | 0.19929428 | -0.41212699 | 1.01748549 | -3.70171401 | -1.4137476 | -0.32920564 |
| **(8) T80-L-2** | -1.07123531 | -0.71357947 | 0.70347121 | -3.0130421 | -4.83658629 | -1.60368885 | -1.98289264 |
| **(9) T80-L-3** | -0.50540807 | -1.58043871 | 1.13276217 | 0.7606232 | -3.83894803 | -2.5757638 | -1.29282929 |
| **(10) T81-L-1** | -1.62170946 | -0.75583676 | 0.77580679 | -3.36809249 | -3.77333922 | -3.30630012 | -2.48197519 |
| **(11) T81-L-2** | -0.38242139 | 1.03817632 | 0.99992063 | 0.36508952 | -4.67018689 | -1.96463638 | -1.50044749 |
| **(12) T81-L-3** | -1.7983322 | 2.04128902 | 0.5954272 | 1.56765176 | -4.55161984 | -1.55775324 | -1.65944671 |
| **(13) T82-L-1** | -2.16680919 | -1.14639484 | -0.40702577 | -4.36735355 | -4.62551509 | -3.65419081 | -2.53498343 |
| **(14) T82-L-2** | -1.22203837 | 2.07963488 | 0.41110346 | -2.73942258 | -6.6042952 | -1.89499992 | -2.63232559 |
| **(15) T82-L-3** | 0.84555191 | 2.15726799 | 1.46874438 | -2.43252172 | -4.16669454 | -0.71003141 | -1.15465027 |
| **(16) T83-L-1** | -0.62804806 | 2.59412373 | -0.50653406 | -2.28589006 | -6.86328454 | -1.06760291 | -2.32201906 |
| **(17) T83-L-2** | -2.83977159 | 0.76931222 | -0.46911335 | -0.38379701 | -4.78496202 | -4.14366881 | -3.37632211 |
| **(18) T83-L-3** | -2.59142029 | 1.28564028 | 0.7591542 | -2.660905 | -5.12961813 | -2.6819545 | -2.37945728 |
| **(19) T85-L-1** | -0.5065714 | 1.38095352 | 0.12419515 | 1.44769928 | -7.14431885 | -0.58521266 | -2.44413879 |
| **(20) T85-L-2** | -1.81329072 | -0.71907914 | 1.34488952 | -3.445648 | -4.74222418 | -2.87524394 | -2.09531001 |
| **(21) T85-L-3** | -2.52097567 | 0.4814562 | 0.72161046 | 1.60853711 | -5.69273814 | -2.91660301 | -3.26605471 |
| **(22) T86-L-1** | -3.77775467 | -1.09355088 | 0.16416593 | -3.87625842 | -6.72981219 | -2.4748658 | -2.73033162 |
| **(23) T86-L-2** | -2.30344568 | 1.80382996 | -0.88103154 | 1.75461846 | -6.66532948 | -2.90981979 | -2.8719036 |
| **(24) T86-L-3** | -3.13471672 | 1.34766001 | -0.85893636 | -5.07339928 | -7.21460538 | -2.70709933 | -3.9246566 |
| **(25) T87-L-1** | -2.31488622 | 0.44576442 | 0.31407344 | -1.02986412 | -6.40109075 | -3.76604983 | -3.19550654 |
| **(26) T87-L-2** | -2.558001 | 0.16020445 | -0.41637337 | -1.53618666 | -6.15258515 | -3.64848372 | -2.78020267 |
| **(27) T87-L-3** | -2.16905385 | -0.17311014 | -0.58897191 | -4.86516426 | -6.58679181 | -3.82358215 | -2.58675048 |
| **(28) T89-L-1** | -1.2860373 | 0.64170852 | 0.50662691 | -3.8314856 | -6.57517101 | -0.97830059 | -1.8754058 |
| **(29) T89-L-2** | -1.53426718 | 0.06563116 | -0.03923646 | -0.27414424 | -4.26850485 | -3.84100889 | -2.64159941 |
| **(30) T89-L-3** | -1.85736697 | 0.35539014 | -0.03829234 | -4.71090199 | -2.77861255 | -3.65418412 | -1.99135504 |
